# Supplementary material for: Bosminopsis deitersi (Crustacea: Cladocera) as an ancient species group: a revision
Source: PeerJ. 2021 Apr 23;9:e11310. doi: 10.7717/peerj.11310 (PMC8074845; doi:10.7717/peerj.11310)
Supplement: Supplemental Information 2 [file peerj-09-11310-s002.docx]

| **Locus** | **Position** | **Best model (BIC)**  ***Bosminopsis* only** | **Best model (BIC)**  ***Bosminopsis* + outgroups** |
| --- | --- | --- | --- |
| COI | 1st | TNe{1.8573,14.8352}  +FQ | TIM3e{0.0001,1.1171,14.1711}  +FQ  +I{0.5705} |
|  | 2nd | TNe{1.8573,14.8352}  +FQ | K3Pu{33.76,100}  +F{0.0941,0.2602,0.1902,0.4554} |
|  | 3th | TNe{1.8573,14.8352}  +FQ | TPM3u{0.2111,5.6469}  +F{0.3029,0.1587,0.1199,0.4182}  +R2{0.57,0.26,0.42,1.97} |
| 16S |  | TVM{7.95,83.89,84.28,2.93}  F{0.3123,0.1436,0.2061,0.3379} +R2{0.83,0.17,0.16,5.25} | TVM{0.72,6.01,5.05,0.33}  F{0.3081,0.1471,0.2061,0.3388} +G4{0.3868} |
| 18S |  | JC  +I{0.8431} | K2P{2.2143}  +FQ  +R2{0.88,0.28,0.11,6.27} |
| 28S |  | K2P{6.9781}  +FQ  +I{0.7354} | TNe{3.7143,12.5595}  +FQ  +G4{0.1927} |

Base substitution rates: JC – Jukes-Cantor model [1] equal base frequencies, all substitutions equally likely; K2P – Kimura 2-parameter model [2] equal base frequencies, one transition rate and one transversion rate; TNe – Tamura-Nei model [3] equal base frequencies, equal transversion rates, variable transition rates; K3Pu – Kimura 3-parameter model [4] unequal base frequencies, equal transition rates, two transversion rates; TIM3e – transversion model with equal base frequencies and AC=CG, AT=GT; TPM3u – three parameter model with unequal base frequencies and AC=CG, AG=CT, AT=GT; TVM – transversion model with variable base frequencies, variable transversion rates, transition rates equal. Base frequencies: +F – empirical base frequencies; +FQ – equal base frequencies. Rate heterogeneity across sites: +I – proportion of invariable sites; +G4 – discrete Gamma model [5] with four categories; +R – FreeRate model [6] that generalizes the +G model by relaxing the assumption of Gamma-distributed rates. Non-standard model parameters are indicated in {}.

References

1. Jukes TH, Cantor CR. Evolution of protein molecules. In: Munro HN, editor. Mammalian protein metabolism. New York: Academic Press; 1969. p. 21–132. doi:10.1016/B978-1-4832-3211-9.50009-7.

2. Kimura M. A simple method for estimating evolutionary rates of base substitutions through comparative studies of nucleotide sequences. J Mol Evol. 1980;16:111–20.

3. Tamura K, Nei M. Estimation of the number of nucleotide substitutions in the control region of mitochondrial DNA in humans and chimpanzees. Mol Biol Evol. 1993;10:512–26. doi:10.1093/oxfordjournals.molbev.a040023.

4. Kimura M. Estimation of evolutionary distances between homologous nucleotide sequences. Proc Natl Acad Sci U S A. 1981;78:454–8.

5. Yang Z. Maximum likelihood phylogenetic estimation from DNA sequences with variable rates over sites: Approximate methods. J Mol Evol. 1994;39:306–14. doi:10.1007/BF00160154.

6. Soubrier J, Steel M, Lee MSY, Der Sarkissian C, Guindon S, Ho SYW, Cooper A. The influence of rate heterogeneity among sites on the time dependence of molecular rates. Mol Biol Evol. 2012;29:3345–58. doi:10.1093/molbev/mss140.
