# Supplementary figures and images for: Bosminopsis deitersi (Crustacea: Cladocera) as an ancient species group: a revision
Source: PeerJ. 2021 Apr 23;9:e11310. doi: 10.7717/peerj.11310 (PMC8074845; doi:10.7717/peerj.11310)

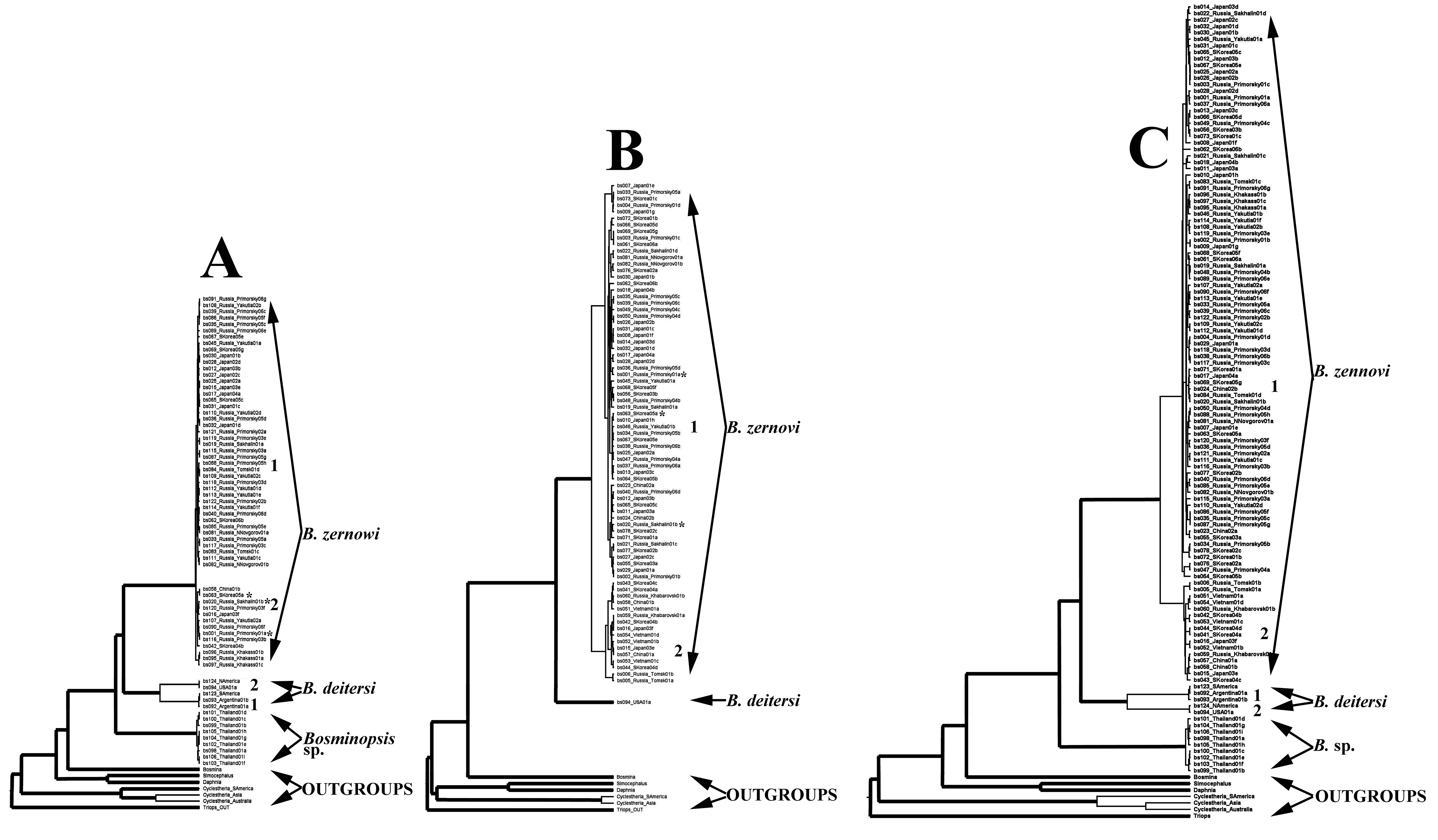

Supplement: Supplemental Information 3 — Branches with support more than 0.7 are bold, while with lower support - thin. Asterisks mark conflicts where taxa were placed in subclade 1 in the mitochondrial tree and subclade 2 in nuclear tree. [file peerj-09-11310-s003.png]
